# “Because We Are Afraid”: voices of the undocumented in a new immigrant destination in the United States

**DOI:** 10.1057/s41271-024-00475-4

**Published:** 2024-04-03

**Authors:** Madeline Metcalf, Danika Comey, Deborah Hines, Genesis Chavez-Reyes, Sally Moyce

**Affiliations:** 1 College of Letters and Science, Montana State University, P.O. Box 172360, Bozeman, MT 59717 USA; 2 Mark and Robyn Jones College of Nursing, Montana State University, Anna P. Sherrick Hall Room 204, Bozeman, MT 59717 USA; 3 South North Nexus, P.O. Box 404, Bozeman, MT 59771 USA; 4 Mark and Robyn Jones College of Nursing, Montana State University, Anna P. Sherrick Hall Room 210, Bozeman, MT 59717 USA

**Keywords:** Undocumented, Immigration, Qualitative, Anti-immigrant policies, Challenges

## Abstract

The purpose of this study is to explore immigrants’ perceptions of their daily lives in a state with anti-immigrant policies in the United States. Using snowball sampling, researchers recruited a sample of 30 Latino immigrants in southwest Montana. The research team conducted semi-structured interviews in Spanish and analyzed the data using thematic analysis. We identified four themes: difficulty accessing healthcare, frustration over the inability to obtain driver’s licenses, challenges related to employment, and desire to make a life in Montana. Fear permeated all topics. Lack of documentation presents complex economic, health, and social challenges that prevent immigrants from fully integrating into their communities. These are exacerbated in states that employ anti-immigrant policies. As Western states continue to experience growth in immigrant populations, it is critical to develop policies to support integration and equitable access to health and social services.

## Key messages

- There are very little data about the health needs of the growing Latino immigrant community in the American West.
- Despite fear and challenges noted by immigrants, most wanted to build their lives in Montana.
- With the growing immigrant population, understanding how documentation status affects daily life is critical for public health professionals in creating inclusive policies and programs.

## Introduction

Nearly a quarter of all immigrants living in the United States (US) lack legal authorization and immigrants from Mexico and Central America account for 67% of the total undocumented population [1, 2]. The US Hispanic population has increased steadily over the past decade. Passel, Lopez, and Cohn categorize Southwest Montana as a new immigrant destination [3], and Tolbert observes it is among places with an immigrant population of less than 5% but growing at a high rate [4]. New immigrant destinations are more likely to have a larger proportion of undocumented immigrants, which presents specific challenges related to an undeveloped infrastructure, such as healthcare systems and social service agencies, without culturally and linguistically appropriate resources [5, 6]. The lack of translated documents, interpreter services, and appropriate referrals for medical care, combined with exclusionary or criminalizing immigration state policies, may make life difficult for undocumented immigrants and may impact their ability to acculturate or participate in the mainstream culture [7, 8]. Concerns about deportation, anti-immigrant sentiments, fear of family separation, and exploitation from a lack of government protections prevent undocumented immigrants from accessing existing services [8–10].

In Montana, a rural state that borders Canada, a string of anti-immigrant policies includes the banning of sanctuary cities, exclusion of immigrants from state licensure, and exclusion from social service programs. Recently, Montana began to participate in a federal program implemented by US Immigration and Customs Enforcement (ICE) known as “287(g)”[11–14]. The aim is to have local and state law enforcement aid ICE to “identify and remove” undocumented individuals from the United States. In 2020, Gallatin County, Montana implemented the Warrant Service Officer Model, part of 287(g), which trains and certifies local law enforcement to perform roles of ICE [11].

Despite these policies, Montana has a longstanding history of Latino migration for economic opportunities. In 1942, an executive order established the Bracero Program, which permitted Mexican men to work in the United States, and in Montana, on short-term labor contracts in response to the agricultural labor shortage [15]. While the United States has since terminated this program, immigrants continue to arrive in Montana and surrounding western states at high rates for employment opportunities within the service, construction, and agriculture industries [16].

Little research focuses on the lived experiences of the undocumented Latino immigrants in new immigrant destinations, particularly in states with anti-immigrant policies. Little knowledge of how these lived experiences impact the lives on immigrants appears in the literature, including their experiences of stress, employment, ability to access services, and the expectations for their futures. We used a community-based participatory research approach to conduct a qualitative study to understand the perceptions of Latino immigrants with or without legal status about how their daily lives are impacted by documentation status in a state with hostile immigrant policies.

## Data and methods

### Study participants and recruitment

The research team recruited a convenience sample of Latino immigrants living and working in southwest Montana via snowball sampling. Specifically, we engaged two Latino community members who approached potential respondents at their places of employment, homes, churches, and eateries. The research team met with interested participants for an interview, conducted in the respondent’s chosen location. Following each interview, we asked respondents to suggest referrals to other potential research participants. We gave each respondent a $25 VISA card in appreciation for his or her time.

### Data collection

We used the Health Belief Model [17] to inform the creation of a semi-structured interview guide to facilitate the collection of qualitative data focused on the lived experiences of immigrant respondents in southwest Montana. We did not ask about the respondents’ immigration status directly. Bilingual and bicultural interviewers, trained in qualitative data collection and the ethical conduct of research, conducted interviews in Spanish between June and October 2021. We audio recorded these interviews, transcribed them verbatim in Spanish, and bilingual members of the research team translated them.

After each interview, the interviewers met to discuss potential bias and assumptions. We also reviewed the interview guide and made necessary changes, based on respondents’ answers. The University’s Institutional Review Board reviewed all study procedures and approved.

### Data analysis

We analyzed the data using NVivo Qualitative Analysis software concurrent with data collection. This allowed for a constant comparison technique and adaptation of the interview guide by the research team. Using thematic coding analysis, we employed an inductive coding methodology to extract themes from the respondent’s answers [18]. The principal investigator assigned a sample of four, randomly selected transcripts to five researchers to review and code independently. All researchers read transcripts for familiarity then identified themes that emerged from transcripts. After we coded the samples, the research team met to discuss themes and created a final codebook that guided the analysis. All members of the research team independently read all transcripts and categorized responses according to the previously defined codebook themes. Consensus meetings and continual assessment of researcher bias allowed us to achieve trustworthiness.

## Results

We conducted interviews with 30 immigrants (19 men and 11 women) before reaching data saturation. We identified four central themes: inability to access healthcare, inability to obtain driver’s licenses, challenges related to employment, and the desire to make a life in Montana (Table 1).

**Table 1:** Quotes from research participants

Theme	Participant quote or definition
Fear	Definition: *Living in fear of deportation or family separation due to not having legal status*
	A policeman can take us prisoner. Every day that we wake up, we go with that thought that it is going to happen or because of a paper, or because we are not residents. We do not have a paper that gives us a permission to be able to be in Montana. So we always go with that panic of any moment—a checkpoint or here you go with speed—and that is the daily fear of not having papers (Participant 5, male)
	I felt, I felt like…like somebody was watching. Like somebody was behind me or something (Participant 8, female)
	The day that they tell me, ‘Look, you have to leave,’ I'm going to leave things abandoned. I don't even have pets, because imagine, I have a pet that I have to leave abandoned and it's not fair and it's not fair to me. So situations like that prevent me from like, like say settling down (Participant 11, male)
	I can't go to my country. Because just like what happened to my brother, it can happen to me. So my fear is that when I arrive they will kill me like they killed my brother. Yes, because he came here and they deported him. And then a week after arriving in my country, they killed him (Participant 11, male)
Access to healthcare	Definition: *Unable to pay for healthcare or find a provider due to lack of insurance, issues with identification and healthcare*
	Because filling out paperwork and everything, and what I don't like is that sometimes in those cases you have to lie. And depending on the…the religion that you have or the teaching that you were given by your parents. You feel bad lying because they force you to lie (Participant 1, male)
	I told you that I don’t like to lie, and I’m being forced to do that on many occasions (Participant 10, female)
Driver’s license	Definition: *Being unable to obtain a driver’s license and the resulting consequences*
	How I wish we had the possibility to get a license from here, from Montana (Participant 8, male)
	Because we are afraid, even driving, because we don’t have a license... there are many fears that we have as immigrants, because we don’t have papers. We don’t have driving papers (Participant 13, female)
	[My license] is not valid. It doesn’t go around, like for an ID, but then the policeman can arrest me for not having a license. He can do it to me (Participant 10, male)
Employment	Definition: *Being paid under the table, receiving less wages than citizens, housing issues related to employer-provided housing*
	We don’t have a work permit—maybe we are working under another name that is not our own. And that makes us tense (Participant 1, male)
	Because in construction, well you can enter to work without documents. Just like in other places, like cleaning, all that. But there are people who take advantage and tell you, ‘Look, I'm going to pay you so much,’ and in the end you come a week later to receive your money and you never hear from the person again. They leave. Well, I say it's a very delicate situation because they shouldn't be like that, because people work out of necessity, and for people who don't have documents who are not paid – it is very hard (Participant 11, male)
	Many times we can call ourselves swindled because they make us work 15 days or a month, and they don’t pay us. These are things that happen, that always happen to us, and [the employers] scare you by telling you immigration is coming (Participant 5, male)
	I have to leave my room and so I stay on the street. It's like suddenly we're…we're like working by force, because if you had the rent…you would look for a better job or you would be treated better (Participant 21, male)
	I’m paid in cash now because I don’t have a way to cash a check. I mean, the employer, many times, he gets annoyed with me because he says he shouldn’t pay me in cash, he wants to pay me by check. But I don’t... I can’t because I can’t access a bank to cash it for me because I don’t have an ID (Participant 4, male)
Hope for the future in Montana	Definition: *A desire to integrate into US culture legally, including a desire to bring family members to US*
	I could say it’s my dream; I love it here (Participant 5, male)
	I would like Montana to be a state that said in the eyes of the law, in the eyes of justice, we are all equal. I moved to Montana, a very nice state, a nice place, and any person feels comfortable. But it would be better if it were equal, equal for everybody (Participant 21, male)
	On immigration, that they be more favorable to us Latinos, so that they give us the opportunity to start a new life here and that they give us the opportunity to get a work permit so that we don’t go around hiding, but that we can work freely and go around honestly (Participant 1, male)
	I lived in Nevada for two years and so I would like [Montana] to be like Nevada: that the immigrant has the same rights as an American (Participant 8, female)

### Fear

Permeating these topics was the theme of fear. Respondents experienced constant fear of deportation for not having legal documentation to live and work in the United States. This created concerns about their health related to persistent psychosocial stress that, for many of them, accompanied the arrival to rural Montana and acculturation. Most often, individuals expressed worry about being deported while they were driving, believing that the lack of a driver’s license would be cause for deportation. The fear extended beyond driving into their everyday life, creating constant distress with potential to impact other areas of health including heart disease, high blood pressure, stroke, among others. This worry prevented many from fully integrating into their new lives in Montana. Fears of deportation included concerns about separation from children or losing everything they had worked for in the United States, though some feared for their lives if they were to be returned to their home country. This constant fear, worry, and stress weighed heavily on participants.

### Access to healthcare

Immigrant respondents described how they were unable to access healthcare due to high costs of care and a lack of health insurance. Despite the comparably high risk of injury in their jobs in construction or in the service sector, employers rarely provided insurance. Respondents specified challenges relating to purchasing private health insurance coverage due to their lack of legal documentation as well as anxiety. They also described their fears, as Latino immigrants, about attempting to use these services. They found it difficult to find health care providers who would see them without insurance. Some noted that they were unable to cover the “high, high costs of a hospital” (participant 12, male), while others feared the healthcare provider would report them to immigration. This often resulted in avoiding healthcare encounters.

A lack of documentation prevented respondents from making use of potentially available financial resources to pay for health services. In the state of Montana, persons without legal authorization to live and work in the US are not permitted to apply for financial assistance from the government or the health facilities, or for payment plans. Respondents told us that they felt compelled to lie about their legal authorization when presented with paperwork to complete. This often conflicted with their moral code, creating a situation where they felt they needed to be dishonest.

### Driver’s license

Respondents repeatedly mentioned the lack of a driver’s license as a challenge, particularly because public transportation in this rural setting is limited. While some respondents had a license from their home country, Montana’s law enforcement does not recognize these licenses. Respondents told us that other states provided means for undocumented individuals to obtain driver’s licenses, but that Montana did not. This barrier created fear in most respondents.

### Employment

Immigrants reported they came to Montana in search of employment opportunities. However, employment also created challenges for respondents. One such challenge was fear for those without legal authorization to work in the United States. Some respondents told us that they chose their jobs based on being able to work without documentation. All respondents reported having employment only in informal jobs without contracts. Working without formal contracts and legal documentation status created fear, anxiety, and worry among the participants. Respondents also noted that they were aware of the precarious situation this placed them in, including being paid less than citizen workers doing the same work. The absence of a contract created opportunities for employers not to pay workers and several respondents mentioned employers seizing this opportunity and not paying their workers.

Respondents mentioned their housing situation as a challenge that tethered them to their jobs because landlords often asked for financial records or for people to pass a background check to ensure no criminal record. Employers, however, sometimes provided housing to their workers, so respondents often lived in employer-provided housing. However, participants noted that if workers leave their jobs, they also lose housing. In addition, lack of legal authorization to live and work in the United States prevented respondents from opening bank accounts or cashing checks, so workers were often paid in cash. Respondents told us their employers often expressed frustration about this method of payment, which was outside of their normal payroll activities.

### Hope for the future in Montana

Despite the fear and the challenges experienced by respondents due to a lack of documentation, all study participants expressed a desire to stay in Montana. Their responses indicate that they were keenly aware of the policies in Montana that prevent them from fully realizing their hopes for a future in the United States.

## Discussion

In this qualitative study, undocumented immigrants described how the lack of legal authorization to live and work in the United States presents complex economic, health, and social challenges that created stress and prevented full integration into a new community, such as being able to find stable housing or secure a driver’s license or navigate social and health services. These challenges are exacerbated in states such as Montana that employ anti-immigrant and exclusionary public policies in combination with an undeveloped infrastructure to support growing minority populations, including lack of translation services and interpreters, challenges getting to clinics, and inconvenient office hours [19]. Respondents overwhelmingly noted that the inability to live and work legally in this state created a sense of fear and tension that permeated their daily lives. Continual stress related to documentation status that we identified in the study has negative impacts on immigrant physical and mental health [20]. Previous studies suggest that undocumented Latinos who experience fear due to several forms of minority status (including language, culture, documentation, socio-economic status) are at risk for increased depression and anxiety [21, 22]. This stress is directly related to the criminalization of immigrants in state policies [23]. A previous study associated anti-immigrant policies with decreased healthcare utilization among Hispanics [24], and this is consistent with our results. Respondents reported that they did not seek medical care due to fears related to their immigration status and the inability to obtain health insurance, resulting in immigrants having borne the full costs of care and having delayed or avoided use of healthcare services.

The inability to obtain a driver’s license in Montana differs from sixteen states in the US that currently offer licenses to undocumented immigrants [25]. Policies that provide opportunities for immigrants to obtain licenses improve road safety for all drivers in the community [26]. There is a positive economic impact, as well, as authorized drivers have increased work productivity and work more shifts [27, 28]. Previous research shows that immigrants desire legal driver’s licenses across states and across community types (rural, urban, suburban) and experience the greatest difficulties in rural locations that often lack public transportation [29].

Occupational exploitation of undocumented workers creates risks to personal safety, especially if housing is tied to employment [30]. Respondents in our study pointed to the threat of employers calling immigration officials if workers complained about working conditions. Immigration raids perpetuated by employers in other locations justify workers’ silences about workplace abuses and beliefs that employers would call immigration authorities if workers do not cooperate on their terms. While none of the respondents in our study reported having experienced a raid while employed in Montana, they did highlight the fear of being separated from their families and children should a raid occur. This fear is common among many undocumented workers [31, 32].

## Limitations

While we attempted to gather a representative sample from the immigrant community in the region, the sampling method of using community members may have limited our reach. Therefore, findings cannot be generalizable to the immigrant population in southwest Montana. Although we tried to include the female perspective on being undocumented, the men in our sample made the statements that more effectively illustrated emergent themes and may be overrepresented in the results.

## Conclusions

This is one of the few studies to investigate the impact of undocumented status on the daily lives of immigrants in a state with anti-immigration policies. We also present information about immigrants’ fears and barriers which impact health outcomes. New immigrant communities contribute to the social and economic vitality of diminishing rural populations, and despite the challenges noted by respondents, most wanted to build their lives in Montana. As rural western states such as Montana continue to experience growth in minority and immigrant populations, public policies must be developed and adapted to support integration and equitable access to necessary health and social services.

## Data Availability

The data that support the findings of this study are available from the corresponding author upon reasonable request.
